# Injectable alginate composite hydrogel with spatiotemporal codelivery of pro-angiogenic and anti-fibrotic agents for synergistic myocardial repair

**DOI:** 10.1016/j.mtbio.2026.102854

**Published:** 2026-01-28

**Authors:** Yujuan Jia, Tongtong Yin, Zhu Wang, Lei Chen, Hongyou Fan, Jiangbin Wu, Qian Yu, Yanxia Zhang, Zhenya Shen

**Affiliations:** a Department of Cardiovascular Surgery of the First Affiliated Hospital & Institute for Cardiovascular Science, Suzhou Medical College of Soochow University, Soochow University, Suzhou, 215006, China; b State and Local Joint Engineering Laboratory for Novel Functional Polymeric Materials, College of Chemistry, Chemical Engineering and Materials Science, Soochow University, Suzhou, 215123, China; c Department of Cardiovascular Surgery of the Qinghai Provincial People's Hospital, Xining, 810000, China

**Keywords:** Myocardial infarction, UCL-TRO-1938, Angiogenesis, Bone morphogenetic protein-9, Antifibrosis

## Abstract

Myocardial infarction (MI), a leading cause of heart failure, involves dynamic pathological progression from acute ischemia to maladaptive fibrosis. To address this complexity, we engineered an injectable alginate composite hydrogel enabling spatiotemporal codelivery of dual therapeutics targeting distinct MI phases. The system incorporates: (i) UCL-TRO-1938, a newly identified PI3Kα activator promoting angiogenesis via PI3K/Akt signaling, released immediately during the acute injury phase; and (ii) engineered mesoporous silica nanoparticles encapsulating bone morphogenetic protein-9 (BMP-9); these nanoparticles feature an epigallocatechin gallate/zinc ion complex coating enabling pH-responsive payload release specifically within acidic infarct microenvironments. This design aims to align the release of UCL-TRO-1938 with the early demands of angiogenesis and delay BMP-9 release to coincide with the later phase of fibrosis progression. Comparative studies in murine myocardial infarction models showed that this dual-delivery platform resulted in improved outcomes compared with single-agent therapies. Intramyocardial administration significantly reduced apoptosis, enhanced angiogenesis, attenuated fibrosis, and improved cardiac function relative to controls. By synchronizing material properties with stage-specific biological responses, this temporally programmed strategy, which aligns with the pathological progression of MI, achieves enhanced functional recovery compared to conventional monotherapies, providing a clinically viable approach for myocardial repair.

## Graphical abstract

**Figure:**
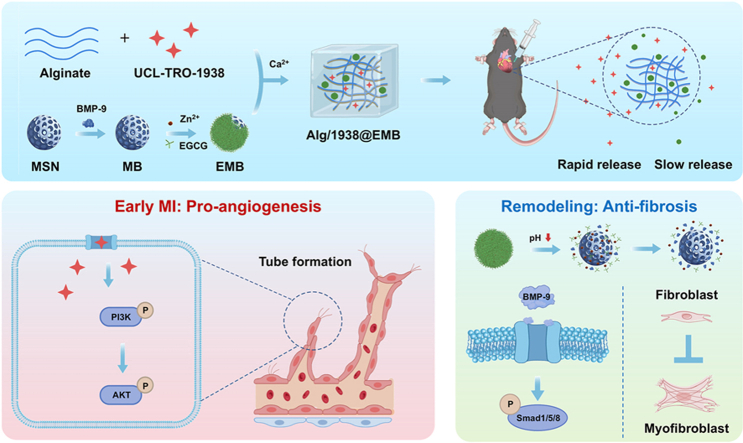

## 1 Introduction

Myocardial infarction (MI), one of the most prevalent cardiovascular diseases, remains a significant global health challenge due to its high morbidity and mortality [1]. Primarily caused by coronary artery occlusion, MI leads to irreversible cardiomyocyte loss, fibrotic scar formation, ventricular wall thinning, and ultimately, adverse left ventricular remodeling and heart failure [2,3]. Current clinical treatments, including pharmacological therapy, coronary artery bypass grafting (CABG), and percutaneous coronary intervention (PCI), primarily focus on restoring perfusion and alleviating acute symptoms [4,5]. However, such treatments have limited potential to reverse the ischemic microenvironment imbalance, or preventing long-term structural and functional deterioration. In addition to re-establishing blood supply, combinatorial strategies such as cardiomyocyte replacement [6], immunomodulatory interventions [7], or anti-fibrotic therapies [8], have shown promise in improving cardiac function. Therefore, therapeutic approaches that align with the pathological progression of MI by stimulating angiogenesis in the early phase and effectively suppressing fibrosis during the later stages hold great promise for facilitating comprehensive structural and functional cardiac repair.

Therapeutic angiogenesis, which aims to promote neovascularization in ischemic myocardium, represents a promising strategy for enhancing myocardial perfusion and supporting tissue regeneration [9]. This approach is often pursued through the targeted delivery of pro-angiogenic agents to stimulate collateral vessel formation. Although gene therapy [10], cell transplantation [11], and protein-based growth factor delivery [12] have shown therapeutic potential, their clinical translation remains limited by challenges such as poor targeting efficiency, bioactivity instability, and difficulties in large-scale production. In contrast, small molecules offer distinct advantages, including high target selectivity, controlled modulation of signaling pathways, favorable pharmacokinetics (e.g., tissue permeability and bioavailability), synthetic accessibility, and low immunogenicity [13,14]. For example, the small molecule TGP-377, which modulates microRNAs regulating VEGFA expression, has demonstrated robust pro-angiogenic effects [15]. Recently, Gong et al. identified a novel small-molecule subtype-selective PI3Kα activator, UCL-TRO-1938, which conferred cardioprotective effects in a murine ischemia–reperfusion model [16]. Given the essential role of the PI3K/AKT signaling pathway in angiogenesis [17], we hypothesized that UCL-TRO-1938 may promote angiogenesis by directly activating PI3K signaling in endothelial cells.

Post-MI fibrotic remodeling is marked by the activation of fibroblasts and their differentiation into myofibroblasts, which excessively secrete extracellular matrix (ECM) components and drive the formation of collagen-rich scar tissue. This process is primarily mediated through the TGF-β/Smad2/3 signaling axis [18,19]. Conversely, bone morphogenetic protein (BMP) signaling, particularly via Smad1/5/8, counteracts fibrosis [20]. Among BMP family members, BMP-9 has emerged as a potent anti-fibrotic cytokine in the heart. Recombinant human BMP-9 (rhBMP-9) has been shown to attenuate cardiac fibrosis and improve cardiac function in preclinical models [21]. However, its therapeutic application is limited by poor pharmacokinetics, rapid degradation, and the requirement for repeated administration. Therefore, developing stable and controlled delivery systems is critical [22]. Mesoporous silica nanoparticles (MSNs) represent attractive carriers for protein therapeutics due to their high surface area, tunable pore structure, biocompatibility, and straightforward surface modification [[23], [24], [25]]. Functionalization with charged groups facilitates strong electrostatic interactions with proteins, enhancing loading efficiency and prolonging release [26,27]. For instance, encapsulating BMP-4 within MSNs allows for sustained protein release, and when incorporated into an injectable double network hydrogel, this system enables prolonged BMP-4 delivery, effectively promoting skull defect repair through controlled release kinetics [28].

However, direct intramyocardial injection of small molecule drugs or MSNs often results in poor retention and rapid clearance due to mechanical washout and the harsh ischemic-inflammatory microenvironment within the infarct zone, thereby limiting therapeutic efficacy. Biomaterial-based local delivery systems offer a viable solution by enhancing agent retention in the infarcted area [29,30]. Injectable hydrogels could be an attractive delivery platform due to their biocompatibility, ECM-mimicking mechanical support, and capacity for localized, sustained, and stimuli-responsive drug release [[31], [32], [33], [34]]. For example, alginate-based hydrogels have been extensively explored in cardiac tissue engineering and have shown promising clinical potential for MI therapy [[35], [36], [37]] Specifically, integrating alginate hydrogels as a primary carrier with silk fibroin microspheres as a secondary carrier enables sequential controlled delivery of dual bioactive molecules to achieve comprehensive therapeutic effects for MI treatment [38].

Given the distinct temporal pathological phases following MI, characterized by early ischemic injury and subsequent fibrotic remodeling, a sequential drug delivery system designed to align with these temporal changes may enhance therapeutic efficacy. Here, we propose a pH responsive alginate hydrogel system that can sequentially release the pro-angiogenic small molecule UCL-TRO-1938 and the anti-fibrotic protein BMP-9. Specifically, BMP-9 is encapsulated in a secondary carrier of MSNs coated with an epigallocatechin gallate/zinc ion (EGCG/Zn^2+^) complex layer (termed EMB NPs), while UCL-TRO-1938 is directly embedded in the primary carrier of hydrogel matrix. The EGCG/Zn^2+^ layer serves dual functions: protecting against premature BMP-9 release and enabling pH-responsive liberation in the acidic infarct microenvironment [39]. This design facilitates rapid UCL-TRO-1938 release to promote early-phase angiogenesis, followed by sustained BMP-9 delivery to mitigate later fibrotic remodeling. The in vitro pro-angiogenic activity of UCL-TRO-1938 and its underlying mechanism in angiogenesis, as well as the anti-fibrotic efficacy of EMB NPs, were characterized using multiple assays. The therapeutic effects of the composite hydrogel on post-MI inflammation, cardiomyocyte apoptosis, angiogenesis, myocardial fibrosis, and cardiac function were subsequently evaluated through comprehensive in vivo assays.

## 2 Experiments

### 2.1 The effect and underlying mechanism of UCL-TRO-1938 on angiogenesis

The bioactivity of UCL-TRO-1938 was evaluated using human umbilical vein endothelial cells (HUVECs). For all subsequent experiments, HUVECs were seeded at appropriate densities and cultured to ∼80 % confluence, followed by serum starvation for 4 h in basal medium to minimize basal signaling activity and synchronize the cells, thereby enabling a more consistent response to subsequent treatments. To mimic the ischemic and hypoxic microenvironment of MI, cells were then incubated under hypoxic conditions (1 % O_2_, 5 % CO_2_, 94 % N_2_) at 37 °C in serum-free endothelial cell medium (ECM) containing the specified concentration of UCL-TRO-1938.

**Cell viability assay**. HUVECs were seeded into 96-well plates (∼5 × 10^3^ cells/well) and cultured for 24 h. After serum starvation and hypoxic treatment with varying concentrations of UCL-TRO-1938 (0, 1, 3, 5, 10, 15, 20, and 25 μM) for 24 h, 10 μL of CCK-8 solution was added to each well at pre-specified time points. The cells were then incubated for an additional 2 h at 37 °C. Subsequently, the absorbance was measured at 450 nm.

**Wound healing assay.** HUVECs were seeded in 6-well plates at a density of 1 × 10^6^ cells per well. Once the cells reached over 80 % confluence, they were serum-starved, and a straight-line “wound” was created by scraping the cell monolayer with a sterile pipette tip. The cells were then incubated under hypoxic conditions in serum-free ECM containing 10 μM UCL-TRO-1938. After 24 h, cell migration into the scratch area was examined under an optical microscope (Olympus, Japan), and the migration rate was quantified using ImageJ software.

**Tube formation assay.** HUVECs were serum-starved, trypsinized and seeded onto Matrigel-coated wells at a density of 3 × 10^4^ cells per well. The cells were then cultured in serum-free ECM containing 10 μM UCL-TRO-1938 under hypoxic conditions at 37 °C for 6 h. Following incubation, capillary-like structures were imaged in five randomly selected fields per well using an optical microscope (Olympus IX51, Japan). For each sample, more than 10 images were randomly taken, and the total tube length was quantified using ImageJ software.

**RNA-seq sample preparation and transcriptomic data analysis.** Total RNA was extracted from HUVECs subjected to hypoxia, with or without UCL-TRO-1938 treatment, using TRIzol reagent. RNA quality and integrity were assessed with the RNA Nano 6000 Assay Kit and an Agilent 2100 Bioanalyzer. Sequencing libraries were constructed via PCR amplification, and their quality was verified using the Bioanalyzer. Qualified libraries were pooled based on effective concentration and expected sequencing depth, then sequenced on an Illumina platform.

Raw RNA-sequencing reads were processed to remove adapter sequences, low-quality reads, and potential contaminants. High-quality reads were aligned to the reference genome, and differential gene expression analysis was conducted using DESeq2 (v1.20.0). To evaluate the potential modulation of angiogenesis-related pathways by UCL-TRO-1938, an angiogenesis-associated gene set (AGGs) was constructed by integrating genes with a GeneCards Relevance Score >3 and genes from the GSEA tumor angiogenesis signature [40]. The overlap between differentially expressed genes (DEGs) and AGGs was visualized using a Venn diagram. Functional enrichment analyses, including Gene Ontology (GO), Kyoto Encyclopedia of Genes and Genomes (KEGG) pathway analysis, and Gene Set Enrichment Analysis (GSEA), were performed to elucidate the biological processes and signaling pathways associated with UCL-TRO-1938 treatment.

### 2.2 The antifibrosis effect of EMB NPs

#### 2.2.1 The preparation of EMB NPs

EMB NPs were synthesized in a two-step process: first, MSNs were dispersed in deionized water (1 mg/mL), sonicated for 3 min, and collected by centrifugation at 9000 rpm before incubation in PBS (pH 7.4) containing BMP-9 (1 μg per mg MSN) at 4 °C with gentle agitation (200 rpm) for 6 h; the resulting BMP-9-loaded nanoparticles (MB NPs) were then collected and washed with deionized water. Next, MB NPs were suspended in 1 mL of Tris buffer (1 M, pH 8.0) and incubated with 100 μL EGCG (10 mg/mL) and 100 μL ZnCl_2_ (equimolar to EGCG) for 3 min, after which the mixture was stirred overnight in the dark to form the EGCG/Zn^2+^ metal–polyphenol network, yielding EMB NPs that were finally collected by centrifugation and washed with deionized water. The encapsulation efficiencies of BMP-9 and EGCG in EMB NPs were 89.5 % and 23.8 %, respectively.

#### 2.2.2 pH release profile of BMP-9 from EMB NPs

To evaluate the pH-responsive release behavior of EMB NPs, 1 mg of EMB NPs was suspended in 1 mL of PBS (pH 5.5 or 7.4) and incubated at 37 °C with constant shaking (120 rpm). At predetermined time points, 100 μL of supernatant was withdrawn and replaced with an equal volume of fresh PBS. BMP-9 concentrations in the collected supernatants were determined by ELISA. All experiments were conducted in triplicate.

#### 2.2.3 Antifibrotic effects of EBM NPs

NIH3T3 cells and primary cardiac fibroblasts (CFs) were used to study the antifibrotic effects of EBM NPs. Cells were cultured in DMEM/F12 supplemented with 10 % fetal bovine serum (FBS) and 1 % penicillin–streptomycin at 37 °C in 5 % CO_2_. For the following experiments, cells were serum-starved in serum-free DMEM/F12 for 4 h to synchronize the cells. To induce myofibroblast differentiation and detect the antifibrotic effect of different NPs, cells were exposed to 10 ng/mL TGF-β for 24 h, either alone or in combination with the following conditions: (i) PBS (control), (ii) EM NPs, (iii) free BMP-9, or (iv) EMB NPs. Final concentrations were 11.2 μg/mL for NPs and 10 ng/mL for BMP-9. Untreated cells without TGF-β1 stimulation served as the negative control.

**EdU proliferation assay.** NIH3T3 cells were seeded at 3 × 10^4^ cells/well in 24-well plates and cultured until ∼70 % confluence. After serum starvation, TGF-β stimulation, and NPs treatment, cells were incubated with EdU according to the Click-iT™ EdU Imaging Kit protocol. Following EdU staining, nuclei were counterstained with DAPI for 10 min. Fluorescence images were captured using an inverted fluorescence microscope (Olympus IX51), and the percentage of EdU-positive cells was quantified using ImageJ software.

**Wound healing migration assay.** NIH3T3 cells were seeded at 5 × 10^5^ cells/well in 6-well plates and cultured to ∼70 % confluence. A “wound” scratch was created across the cell monolayer using a sterile 200 μL pipette tip. Cells were subsequently subjected to serum starvation, TGF-β stimulation, and NPs treatment. The wound area was imaged at 0 and 24 h after scratching using an inverted microscope. The wound closure percentage was calculated by comparing the wound width at 24 h to that at 0 h using ImageJ.

**Immunofluorescence staining***.* CFs were isolated from 1–3-day-old neonatal C57BL/6 mice by mincing excised hearts followed by enzymatic digestion at 37 °C with 1 mg/mL collagenase II and 0.25 % trypsin. Supernatants were collected every 10 min and pooled. After centrifugation (800 rpm, 5 min), the cell pellets were resuspended and subjected to 1.5 h of differential adhesion for CFs enrichment. Cells were maintained in DMEM/F12 containing 10 % FBS and 1 % penicillin/streptomycin at 37 °C with 5 % CO_2_. Passage 3 CFs were used for subsequent experiments. CFs were seeded in confocal dishes at a density of 1.5 × 10^5^ cells/mL and cultured overnight. After serum starvation, TGF-β stimulation, and NPs treatment, cells were fixed with 4 % paraformaldehyde (PFA), followed by overnight incubation at 4 °C with a primary antibody against α-smooth muscle actin (α-SMA). Cells were then incubated with Alexa Fluor–conjugated secondary antibodies and DAPI for nuclear staining for 1 h at 37 °C in the dark. Fluorescence images were acquired using an Olympus IX51 fluorescence microscope (Japan), and the percentage of α-SMA–positive cells was quantified using ImageJ (NIH, USA).

### 2.3 Preparation and characterization of the Alg/1938@EMB hydrogel

#### 2.3.1 Preparation of the Alg/1938@EMB hydrogel

To prepare the Alg/1938@EMB hydrogel, 1.120 mg of EMB NPs and 1 μL of 10 mM UCL-TRO-1938 solution were added to 500 μL of 1.5 % (w/v) sodium alginate solution under continuous stirring. Subsequently, 500 μL of 0.68 % (w/v) calcium gluconate solution was added dropwise to initiate ionic crosslinking, resulting in the formation of the Alg/1938@EMB hydrogel. For comparison, the hydrogels with only UCL-TRO-1938 or EMB NPs were prepared and used as controls.

#### 2.3.2 In vitro release of UCL-TRO-1938 and BMP-9 from the composite hydrogel

To evaluate the dual release profiles of UCL-TRO-1938 and BMP-9, Alg/1938@EMB hydrogels (1 mL) were incubated in an equal volume of PBS (pH 7.4 or 5.5) at 37 °C under gentle agitation. At predetermined time points, 200 μL PBS was collected and 200 μL fresh PBS was added. The collected PBS was stored at −80 °C for further analysis. UCL-TRO-1938 concentrations were measured by UV–Vis, and BMP-9 levels were quantified using a commercial ELISA. All experiments were performed in triplicate and independently repeated at least three times to ensure reproducibility.

### 2.4 MI mouse model and hydrogel intramyocardial injection

All animal experiments were conducted in accordance with the guidelines approved by the Ethics Committee of the First Affiliated Hospital of Soochow University. Male C57BL/6 mice (6–8 weeks old, 22 ± 2 g) were fasted for 12 h (water withheld) prior to surgery. Under anesthesia, mice were intubated, mechanically ventilated, and continuously monitored by electrocardiogram (ECG). MI was induced by permanently ligating the left anterior descending coronary artery (LAD) ∼2 mm distal to the left atrial appendage using a 6–0 polypropylene suture. Successful infarction was confirmed by the immediate pallor of the anterior ventricular wall and apex, accompanied by ST-segment elevation on the ECG.

Following MI induction, mice were randomly assigned into six experimental groups as listed in Table 1. A total of 10 μL of the respective formulation was then injected into the peri-infarct border zone at four separate sites (2.5 μL per site).

**Table 1:** Description of samples in this work.

Abbreviation	Sample description
Sham	Thoracotomy without ligation
Saline	Saline
Alg	Alg hydrogel
Alg@EMB	Alg hydrogel containing EMB NPs
Alg/1938	Alg hydrogel containing UCL-TRO-1938
Alg/1938@EMB	Alg hydrogel containing UCL-TRO-1938 and EMB NPs

#### 2.4.1 Cardiac function assessment

Cardiac function was evaluated by high-resolution echocardiography (Vevo 2100, VisualSonics, Canada) at baseline and on days 3, 14, and 28 post-MI. Mice were anesthetized and placed on a temperature-controlled platform during imaging. Parasternal long- and short-axis views at the papillary muscle level were acquired using both B-mode and M-mode imaging. Left ventricular ejection fraction (EF) and fractional shortening (FS) were obtained to evaluate systolic function, while left ventricular internal diameters at end-diastole (LVIDd) and end-systole (LVIDs) were calculated to assess ventricular remodeling. All parameters were averaged over three consecutive cardiac cycles.

#### 2.4.2 Histological and immunofluorescence analyses

At specified time points post-MI (day 3 for TUNEL, day 7 for hematoxylin and eosin (H&E), and day 28 for Masson's trichrome and CD31 staining), mice were anesthetized. Hearts were perfused through the aorta with cold cardiac arrest solution (10 % KCl), followed by PBS, and then 4 % paraformaldehyde (PFA).

For cryosectioning (used for TUNEL analysis), hearts were fixed in 4 % PFA at 4 °C for 24 h, then followed by gradient dehydration using a sucrose series (10 %, 20 %, and 30 % in PBS). Samples were embedded in an optimal cutting temperature (OCT) compound and stored at −80 °C. Transverse cryosections (5 μm thick) were cut at 500-μm intervals from the ligation site to the apex using a cryostat (Leica CM1950). For paraffin embedding—used in H&E, Masson's trichrome, and CD31 staining—hearts were dehydrated through graded ethanol (70–100 %), cleared with xylene, infiltrated with paraffin at 60 °C, and then embedded. Serial sections (5 μm thick) were collected every 500 μm using a microtome and stored at 4 °C until use.

**TUNEL staining.** On day 3 post-MI, frozen heart sections were stained using a TUNEL assay kit. Cardiac troponin T (cTnT) labeled cardiomyocytes and DAPI stained nuclei. Apoptotic cardiomyocytes were quantified as the ratio of TUNEL^+^ cTnT^+^ cells/Total cTnT^+^ cells, based on three randomly selected fields per section.

**H&E and Masson staining.** Heart tissue sections were stained with H&E (Solarbio, China) and Masson's trichrome following the manufacturer's protocols. H&E staining was performed on day 7 post-MI surgery to evaluate inflammatory cell infiltration, nuclear morphology, and myocardial fiber integrity. Masson's trichrome staining was performed on day 28 post-MI surgery to assess LV wall thickness and collagen deposition. Fibrotic area was quantified using ImageJ software, and fibrosis extent was expressed as the percentage of fibrotic area relative to the total LV area, calculated using the formula:

Fibrosis(%)=(fibroticarea/totalLVarea)×100%

**CD31 immunofluorescence.** At 28 days post-MI, heart sections were deparaffinized and subjected to antigen retrieval in citrate buffer (pH 6.0, 95 °C, 20 min). Sections were permeabilized with 0.3 % Triton X-100 for 10 min and blocked with 5 % bovine serum albumin for 1 h. To assess neovascularization, cardiac tissue sections were treated overnight at 4 °C with a primary antibody against CD31 (1:200, Abcam, ab28364). Co-staining with an anti-cardiac troponin T (cTnT) antibody (1:300, Abcam, ab8295) was performed to identify cardiomyocyte regions. Following PBS washes, sections were incubated with Alexa Fluor 488-conjugated secondary antibody for CD31 and Alexa Fluor 594-conjugated secondary antibody for cTnT, each for 1 h at room temperature in the dark, and nuclei were counterstained with DAPI. Fluorescence images were acquired from three randomly selected fields per region (border zone and infarct core) using an inverted fluorescence microscope. Vascular density was quantified as the CD31^+^ area relative to the total field area (% CD31^+^ area) using ImageJ.

**Infarct size measurement.** At 28 days post-MI induction, hearts were harvested, rinsed in cold saline, and frozen at −80 °C. The frozen hearts were then transversely sectioned into 1 mm-thick slices. Heart sections were incubated in 2 % 2,3,5-triphenyltetrazolium chloride (TTC) solution at 37 °C for 30 min in the dark to distinguish viable from infarcted tissue. After staining, the sections were fixed in 4 % PFA. Viable myocardium was stained red, while infarcted areas appeared white. Images were captured using a stereomicroscope (Nikon, China), and infarct size was quantified using ImageJ software.

## 3 Results and discussion

### 3.1 Promotion of angiogenesis by UCL-TRO-1938 in vitro

Ischemic injury in the infarcted myocardium constitutes a primary driver of cardiomyocyte loss and subsequent cardiac dysfunction, establishing revascularization as a critical therapeutic objective post-MI [41]. Angiogenesis is orchestrated through key signaling pathways, with PI3K/AKT playing a central role in regulating endothelial cell proliferation, migration, and angiogenesis [42]. Consequently, therapeutic modulation of PI3K/AKT signaling represents a promising strategy to attenuate adverse post-MI remodeling [43]. Recent studies identified UCL-TRO-1938, a novel subtype-selective PI3Kα activator, demonstrating transient PI3K activation and cardioprotective effects in murine ischemia-reperfusion models [16]. Given the pivotal role of PI3K/AKT in angiogenesis and the hypoxic microenvironment following MI, we hypothesized that UCL-TRO-1938 promotes endothelial angiogenic activity under hypoxic conditions, suggesting therapeutic potential for MI. To investigate this, we assessed UCL-TRO-1938 effects on HUVECs viability and angiogenic function under hypoxia. Preliminary analysis revealed dose-dependent enhancement of HUVECs viability, plateauing at 10 μM (Fig. S1); this concentration was consequently selected for subsequent evaluations. Hypoxic conditions reduced viability to 65 % of normoxic levels, while UCL-TRO-1938 treatment partially restored viability to approximately 85 % (Fig. S2). Angiogenic capacity was further evaluated through migration and tube formation assays.

**Fig. 1:**
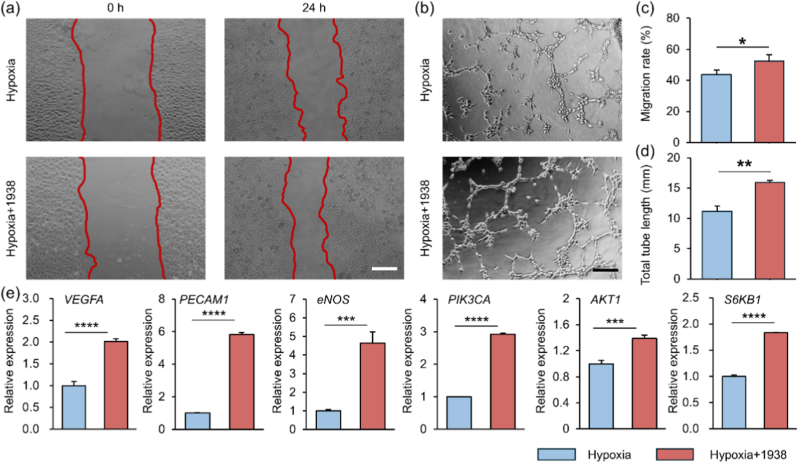
Effect of UCL-TRO-1938 on HUVECs under hypoxic conditions. (a) Representative bright-field images (Scale bar: 200 μm) and (c) quantitative analysis of HUVECs migration. (b) Representative images (Scale bar: 200 μm) and (d) quantitative analysis of total tube length in HUVECs tube formation assays. (e) qRT-PCR analysis of mRNA expression levels of angiogenesis-related genes (VEGFA, PECAM1, eNOS, PIK3CA, AKT1, S6KB1) in HUVECs under hypoxia. Data are mean ± SD (*n* = 3; ∗*p* < 0.05, ∗∗*p* < 0.01, ∗∗∗*p* < 0.001, ∗∗∗∗*p* < 0.0001).

Scratch assays showed that UCL-TRO-1938 significantly enhanced HUVECs migration under hypoxia (52.6 ± 3.9 % vs. 43.7 ± 3.1 %; Fig. 1a–c). In tube formation assays, treated cells formed more branch nodes and mesh structures compared to the fragmented networks seen in controls (Fig. 1b–d). qRT-PCR analysis revealed that UCL-TRO-1938 upregulated angiogenic genes (VEGFA, eNOS, PECAM1) and PI3K/AKT pathway components (PIK3CA, AKT1, S6KB1) relative to hypoxic controls (Fig. 1e), consistent with PI3K/AKT pathway activation. These results align with recent findings that UCL-TRO-1938 can bypass surface receptors to directly activate PI3Kα in murine models, boosting eNOS expression and reducing infarct size in insulin-resistant MI mice [44]. Together, these data indicate that UCL-TRO-1938 promotes endothelial viability, migration, and network formation under hypoxia via PI3K/AKT signaling.

**Fig. 2:**
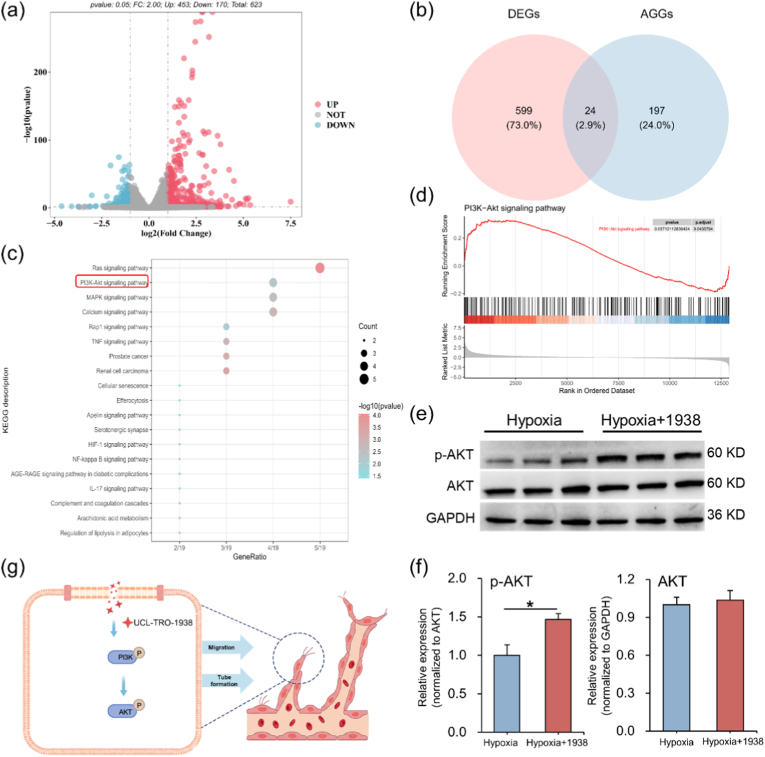
UCL-TRO-1938 protects HUVECs under hypoxia via PI3K/AKT pathway activation. (a) Volcano plot of DEGs between Hypoxia+1938 and Hypoxia groups (|log_2_FC| > 1, *p* < 0.05). (b) Venn diagram showing the overlap between DEGs and AGGs. (c) KEGG pathway enrichment analysis identified 19 significantly enriched pathways. Among them, six were directly associated with angiogenesis, including Ras, PI3K-Akt, TNF, MAPK, HIF-1, and Apelin signaling pathways. (d) GSEA confirmed significant activation of the PI3K-Akt signaling pathway. (e) Western blot analysis showing increased AKT phosphorylation following UCL-TRO-1938 treatment. (f) Quantification of Western blot results shown in (e). (g) Schematic illustration of UCL-TRO-1938 promoting HUVECs migration and tube formation via activation of the PI3K/AKT pathway.

To further elucidate the protective mechanism of UCL-TRO-1938 under hypoxic conditions, RNA-sequencing was performed on HUVECs. Differential expression analysis using DESeq2 (Hypoxia +1938 vs. Hypoxia) identified 623 differentially expressed genes (DEGs), including 453 upregulated and 170 downregulated genes (Fig. 2a). To specifically assess the effects on angiogenesis, AGGs was compiled by integrating genes with a GeneCards Relevance Score >3 and genes from the GSEA tumor angiogenesis signature [40]. A Venn diagram was constructed to visualize the overlap between DEGs and AGGs. As shown in Figs. 2b and 24 angiogenesis-related DEGs were identified in Hypoxia +1938 versus Hypoxia comparison. KEGG enrichment highlighted six angiogenesis-associated pathways: Ras, PI3K-Akt, TNF, MAPK, HIF-1, and Apelin signaling (Fig. 2c). Gene Set Enrichment Analysis (GSEA) confirmed PI3K-Akt pathway activation (Fig. 2d). Western blotting further demonstrated increased AKT phosphorylation upon UCL-TRO-1938 treatment, corroborating PI3K/Akt involvement (Fig. 2e and f). Together, these results indicate that UCL-TRO-1938 promotes endothelial angiogenesis under hypoxia primarily via PI3K/Akt signaling as shown in Fig. 2g.

### 3.2 Preparation and characterization of EMB NPs

**Fig. 3:**
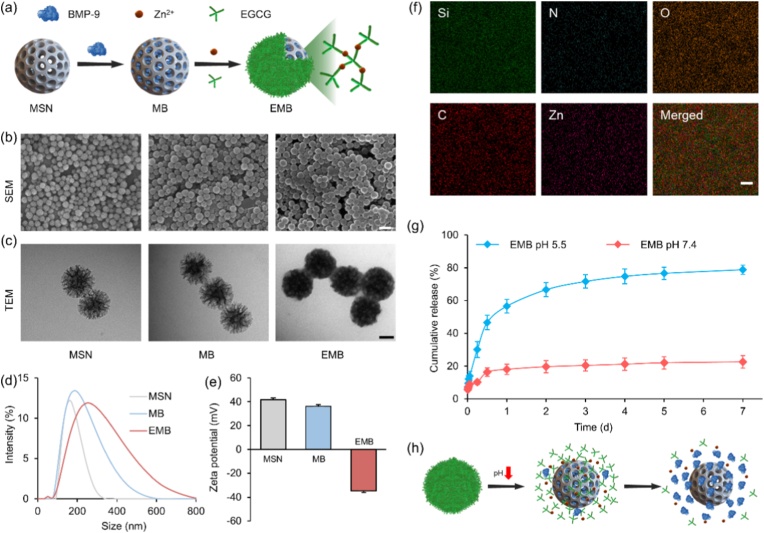
Synthesis, characterization, and responsive release of EMB NPs. (a) Schematic illustration of EMB nanoparticle fabrication. (b) SEM images (Scale bar: 200 nm), (c) TEM images (Scale bar: 50 nm), (c) Size distribution, and (d) Zeta potential of MSN, MB, EMB NPs. (f) EDS elemental mapping of EMB NPs (Scale bar: 0.2 μm). (g) Cumulative BMP-9 release from EMB NPs under different pH conditions. (h) Schematic of pH-responsive BMP-9 release from EMB NPs. Data are mean ± SD (*n* = 3).

Myocardial fibrosis significantly contributes to adverse cardiac remodeling and contractile dysfunction following MI [45].As an endogenous anti-fibrotic cytokine, BMP-9 has demonstrated efficacy in inhibiting fibrosis and attenuating pathological cardiac remodeling [21]. However, the therapeutic potential of systemic BMP-9 administration is constrained by inherent limitations including poor stability and short plasma half-life, necessitating frequent dosing to maintain therapeutic concentrations. Consequently, developing delivery systems enabling microenvironment-responsive release (e.g., pH-triggered mechanisms) could enhance BMP-9 efficacy in MI treatment. Herein, we engineered a hybrid nanoparticle platform (EMB NPs) for this purpose, as illustrated in Fig. 3a. Specifically, BMP-9 was electrostatically loaded onto amine-functionalized MSNs to form MB NPs, followed by coating with an EGCG/Zn^2+^ complex to yield final EMB NPs. This complex coating simultaneously prevents premature BMP-9 release and enables pH-responsive liberation within acidic infarct microenvironments [39]. Importantly, EGCG and Zn^2+^ provide complementary bioactivity: EGCG exerts potent antioxidant and anti-inflammatory effects that mitigate myocardial injury [46], while Zn^2+^ promotes endothelial cell proliferation and migration to support vascular regeneration [47].

A range of characterization techniques was employed to assess the morphology, size, and surface charge of EMB NPs, confirming the effectiveness of the modification process (Fig. 3b–e). Unmodified MSNs exhibited uniform walnut-like morphology with 164.3 ± 2.6 nm diameter and 41.8 ± 1.4 mV zeta potential. BMP-9 loading increased particle size to 190.0 ± 4.5 nm while reducing zeta potential to 36.4 ± 1.4 mV. Subsequent EGCG/Zn^2+^ coating generated a distinct surface layer, further increasing size to 255.0 ± 3.2 nm and reversing zeta potential to −34.6 ± 1.4 mV. In addition, elemental mapping via EDS confirmed homogeneous distribution of Si, C, O, N, and Zn throughout EMB NPs (Fig. 3f), with Zn presence indicating successful EGCG/Zn^2+^ coating. Consistent with established behavior of phenolic-metal complexes, the EGCG/Zn^2+^ coating exhibited pH-dependent dissociation characteristics. We therefore quantified BMP-9 release kinetics from EMB NPs across physiologically relevant pH conditions (Fig. 3g). EMP NPs showed significantly accelerated BMP-9 release at pH 5.5 compared to pH 7.4, consistent with acid-triggered complex dissociation as illustrated in Fig. 3h. Cumulative release reached 78.9 ± 2.8 % during the 7-day evaluation period under acidic conditions (pH 5.5), contrasting with 22.6 ± 3.9 % release observed at physiological pH 7.4. These results collectively establish EMB NPs as an efficient encapsulation platform enabling localized, pH-responsive BMP-9 delivery for targeted anti-fibrotic therapy in MI.

### 3.3 Antifibrotic activity of EMB NPs in vitro

The activation of fibroblasts into myofibroblasts and excessive ECM deposition are hallmark pathological features of myocardial fibrosis, driving scar formation and adverse ventricular remodeling post-MI [45,48]. To assess the antifibrotic effects of EMB NPs in vitro, a TGF-β1–induced fibroblast activation model was established using NIH3T3 cells to mimic the post-MI fibrotic microenvironment. Cells were co-cultured with different NPs under TGF-β1 stimulation. Since fibrosis progression involves myofibroblast proliferation and migration, the effects of EMB NPs on these behaviors were investigated.

**Fig. 4:**
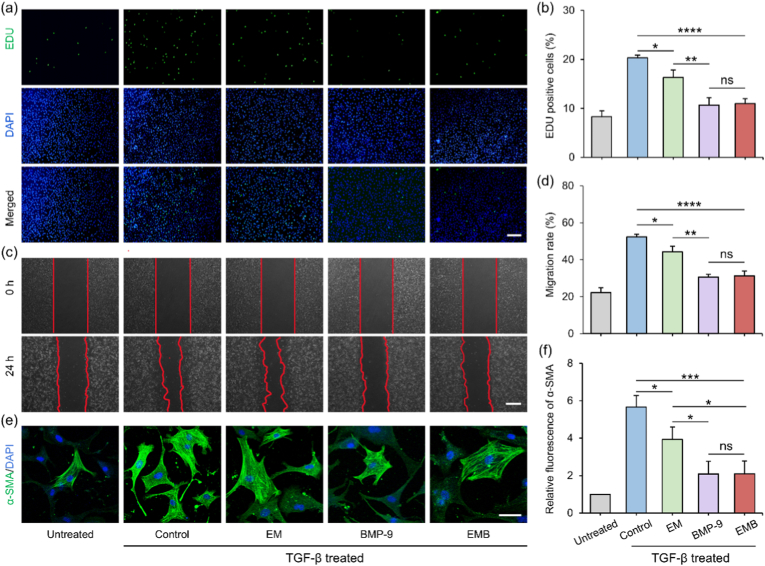
In vitro assessment of antifibrotic activity of EMB NPs. (a) Representative EdU staining images of untreated and treated NIH3T3 (scale bar: 200 μm) and (b) Quantitative analysis of EdU^+^ cells. (c) Scratch assay images showing migration of untreated and treated NIH3T3 over 24 h (scale bar: 500 μm) and (d) Corresponding migration rates. (e) Immunofluorescence images of α-SMA in untreated and treated primary CFs (scale bar: 50 μm) and (f) Quantitative analysis of α-SMA expression. Data are mean ± SD (*n* = 3; ∗*p* < 0.05, ∗∗*p* < 0.01, ∗∗∗*p* < 0.001, ∗∗∗∗*p* < 0.0001).

First, the proliferation of myofibroblasts was calculated by the EdU staining assay. As shown in Fig. 4a and b, TGF-β1 significantly increased EdU-positive nuclei proportion (20.3 ± 0.6 %) compared to PBS controls (8.3 ± 1.2 %). Conversely, EM NPs (EMB NPs without BMP-9), free BMP-9, and EMB NPs treatments reduced cell proliferation to 16.3 ± 1.6 %, 10.7 ± 1.5 %, and 11.0 ± 1.1 %, respectively, demonstrating effective suppression of TGF-β1-induced cell proliferation. EMB NPs and free BMP-9 exhibited comparable efficacy, confirming preserved BMP-9 bioactivity in the EMB NPs sustained-release formulation. Notably, EM NPs lacking BMP-9 also conferred moderate anti-proliferative effects, probably attributable to EGCG's intrinsic anti-fibrotic properties [49]. Wound healing assay results showed a consistent trend, with the EMB NPs group exhibiting the lowest cell migration rate following TGF-β1 treatment (Fig. 4c and d).

To enhance physiological relevance, primary CFs were used (purity: 99.5 %, Fig. S3). Following TGF-β1 stimulation and treatment with different NPs, α-SMA immunofluorescence staining was performed to assess myofibroblast differentiation (Fig. 4e). Quantified results showed that TGF-β1 elevated α-SMA expression 5.7-fold relative to baseline, while EM NPs, BMP-9, and EMB NPs reduced expression to 3.9, 2.1, and 2.1-fold, respectively (Fig. 4f). EMB NPs and BMP-9 groups showed equivalent maximal suppression, further verifying functional BMP-9 preservation. Collectively, EMB NPs exerted potent antifibrotic effects across both fibroblast lines and primary CFs, supporting their therapeutic potential for post-MI fibrosis.

### 3.4 Preparation and characterization of Alg/1938@EMB hydrogel

**Fig. 5:**
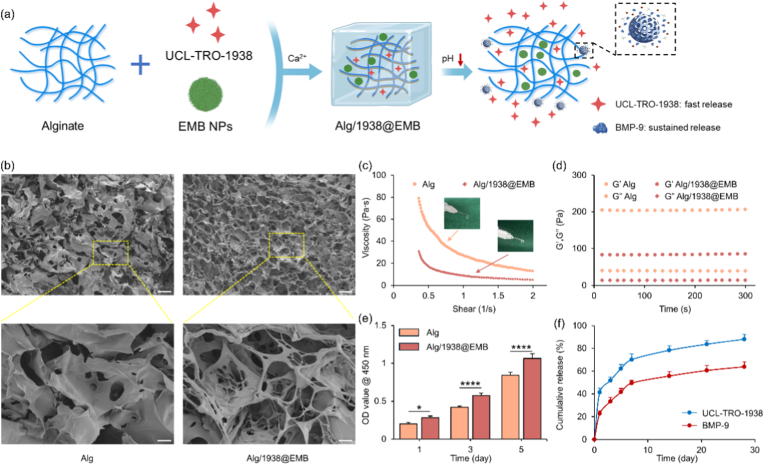
Characterization and therapeutic release behavior of Alg/1938@EMB hydrogels. (a) Schematic workflow for Alg/1938@EMB hydrogel fabrication. (b) Representative SEM images of Alg and Alg/1938@EMB (scale bars: 100 μm; 20 μm for enlarged images). (c) Viscosity profiles of Alg and Alg/1938@EMB hydrogels under varying shear rates. (d) Time-dependent storage (G′) and loss (G″) moduli of Alg and Alg/1938@EMB hydrogels. (e) Proliferation of HUVECs cultured on different hydrogels after different time periods. (f) Cumulative release of UCL-TRO-1938 and BMP-9 from Alg/1938@EMB at pH 5.5. Data are mean ± SD (*n* = 3).

Although small-molecule drugs and nanoplatforms show therapeutic potential for MI, their clinical efficacy remains limited by delivery challenges. Both systemic and intramyocardial administration suffer from rapid drug clearance due to cardiac mechanical motion and high perfusion, hindering sustained therapeutic retention at the infarct site. Alginate hydrogels have emerged as promising reservoirs, with clinical trials validating their safety and efficacy in heart failure [50]. We therefore engineered a composite hydrogel (Alg/1938@EMB) by incorporating UCL-TRO-1938 and EMB NPs into alginate (Fig. 5a), enabling sequential release: initial UCL-TRO-1938 burst release for early angiogenesis, followed by sustained BMP-9 delivery to modulate fibrosis during later remodeling.

As shown in Fig. 5b, the freeze-dried alginate hydrogel possessed a porous sponge-like architecture with uniform structure and smooth surface. In contrast, Alg/1938@EMB maintained interconnected porosity but exhibited more uniform pore distribution, rougher surfaces, and granular features attributed to nanoparticle aggregation. Both hydrogels displayed shear-thinning behavior, allowing injection through 30-gauge needles via viscosity reduction under shear stress (Fig. 5c) with G′ > G″ confirming elastic dominance in rheological testing (Fig. 5d). Cytocompatibility assessments using major cardiac cell types (HL-1 cardiomyocytes, HUVECs, NIH3T3 fibroblasts) showed no toxicity (Fig. S4–S6). Notably, released UCL-TRO-1938 maintained endothelial proliferative capacity (Fig. 5e). In vitro release kinetics under simulated acidic infarct conditions (pH 5.5) demonstrated differential drug release profiles. As shown in Fig. 5f, UCL-TRO-1938 exhibited rapid early-phase release with cumulative percentages of 41.3 ± 3.8 % (Day 1), 70.1 ± 5.1 % (Day 7), and 88.1 ± 4.3 % (Day 28). Conversely, BMP-9 displayed sustained release kinetics matching fibrotic progression rates: 22.7 ± 2.5 % (Day 1), 49.4 ± 2.4 % (Day 7), and 63.7 ± 4.3 % (Day 28). This staged release profile supports MI therapeutic requirements: immediate angiogenesis induction followed by prolonged anti-fibrotic activity.

### 3.5 Therapeutic effects of Alg/1938@EMB in vivo

In vitro studies confirmed the pro-angiogenic activity of UCL-TRO-1938 and antifibrotic efficacy of BMP-9, with the Alg/1938@EMB hydrogel enabling sequential sustained release of both agents. To evaluate in vivo therapeutic efficacy, we established a murine MI model by permanent LAD coronary artery ligation (Fig. S7). The mice were randomly assigned to six experimental groups: Sham, Saline, Alg, Alg@EMB, Alg/1938, and Alg/1938@EMB. The Sham and Saline groups served as negative and positive controls, respectively.

#### 3.5.1 Reducing apoptosis and mitigating inflammation

Ischemic injury induces cardiomyocyte apoptosis within the infarcted area, triggering acute pro-inflammatory responses characterized by the release of cytokines and chemokines. These mediators recruit inflammatory cells to the infarct zone, amplifying inflammatory cascades. Unresolved inflammation exacerbates ischemic damage, promoting interstitial fibrosis and collagen deposition in viable myocardium, ultimately compromising ventricular function [51]. Consequently, attenuating apoptosis and inflammation is critical for effective post-MI repair.

**Fig. 6:**
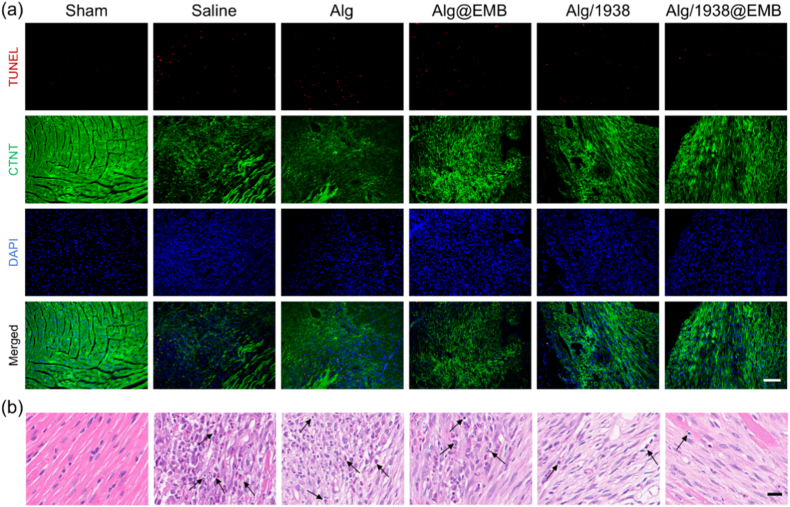
Therapeutic effects of Alg/1938@EMB on cardiomyocyte apoptosis and inflammation post-MI. (a) TUNEL staining showing cardiomyocyte apoptosis in infarcted myocardium (day 3 post-MI; scale bar: 100 μm). (b) H&E-stained sections of infarcted myocardium. (day 7 post-MI; scale bar: 50 μm), black arrow indicates the location of immune cells.

To assess the Alg/1938@EMB hydrogel's therapeutic effects, we quantified cardiomyocyte apoptosis via TUNEL staining (Fig. 6a, Fig. S8) and evaluated inflammatory infiltration within the infarcted myocardium using H&E staining (Fig. 6b). On day 3 post-MI, saline (MI control) and Alg groups exhibited high apoptosis rates (22.0 ± 0.7 % and 21.6 ± 1.1 %, respectively). Alg@EMB and Alg/1938 treatments reduced apoptosis to 19.4 ± 1.5 % and 18.8 ± 1.3 %. Notably, Alg/1938@EMB demonstrated maximal protection (15.6 ± 1.1 %). The anti-apoptotic effect of Alg@EMB may derive from EGCG's antioxidant and anti-inflammatory properties in EMB NPs, known to scavenge ROS and modulate NF-κB/MAPK pathways [52]. The cytoprotective effects of Alg/1938 may be attributed to UCL-TRO-1938–mediated inhibition of intrinsic apoptosis. Additionally, previous studies have reported that UCL-TRO-1938 can protect ischemia-reperfusion–injured myocardium by modulating the IL-17 signaling pathway, thereby reducing IL-17–induced cardiomyocyte apoptosis and neutrophil infiltration [53]. These findings indicate synergistic cardioprotection by EGCG and UCL-TRO-1938 in the composite hydrogel. Moreover, H&E analysis on day 7 post-treatment corroborated anti-inflammatory effects. The Sham group maintained intact myocardial architecture without inflammation, while Saline controls exhibited severe neutrophil infiltration, architectural disruption, and loose collagen deposits. Conversely, the Alg/1938@EMB group preserved tissue integrity with minimal inflammatory infiltration and interstitial damage. Taken together, these findings demonstrate that the Alg/1938@EMB hydrogel effectively modulates the post-infarction microenvironment by reducing cardiomyocyte apoptosis and suppressing inflammatory responses. These combined effects are expected to facilitate improved myocardial preservation and support subsequent cardiac repair following MI.

#### 3.5.2 Promoting angiogenesis

**Fig. 7:**
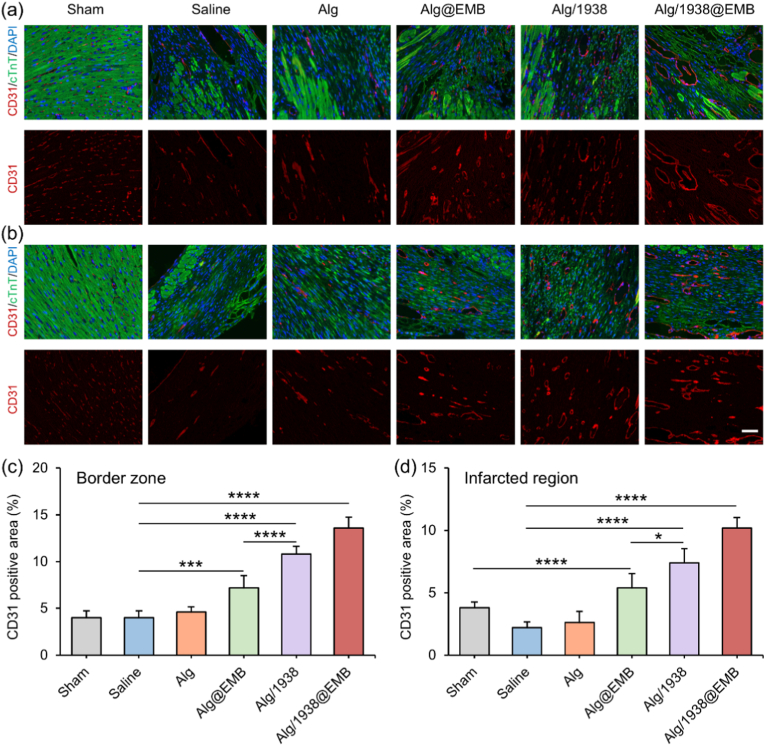
The effects of Alg/1938@EMB on angiogenesis post-MI. (a, b) Representative immunofluorescence images of CD31-stained border zone (a) and infarcted region (b) areas at Day 28 post-MI (scale bar: 50 μm). (c, d) Quantification of vessel density in the corresponding regions. Data are mean ± SD (*n* = 5; ∗*p* < 0.05, ∗∗∗*p* < 0.001, ∗∗∗∗*p* < 0.0001).

MI initiates a defined healing cascade comprising three sequential phases: inflammation, proliferation, and maturation/remodeling [54]. Neovascularization primarily occurs during proliferation, restoring perfusion by delivering oxygen and nutrients to ischemic myocardium [55]. Effective angiogenesis promotion is therefore critical for salvaging viable cardiomyocytes and facilitating functional recovery [56]. To evaluate angiogenic responses, heart tissues harvested at day 28 post-MI underwent immunofluorescence staining of CD31 (an established marker of endothelial cells). Quantification of CD31^+^ vessels in infarcted and border zones was performed to assess regional neovascularization (Fig. 7). Saline and Alg groups exhibited similarly low vessel densities, confirming alginate's minimal intrinsic pro-angiogenic activity—consistent with our previously work [38]. The Alg@EMB group showed moderate neovascularization enhancement in both regions, potentially attributable to: (i) EGCG modulating early inflammation to improve the angiogenic microenvironment [46], and (ii) Zn^2+^ promoting endothelial proliferation and migration [57]. Notably, Alg/1938 and Alg/1938@EMB groups demonstrated significantly higher vascularization than Alg@EMB, validating UCL-TRO-1938's potent pro-angiogenic effect. Among all groups, Alg/1938@EMB achieved maximal vessel density in border and core zones, demonstrating synergistic angiogenic promotion for enhanced tissue perfusion and myocardial repair.

#### 3.5.3 Inhibiting fibrosis

Following MI, cardiac fibroblasts differentiate into myofibroblasts that deposit fibrotic scar tissue to replace necrotic myocardium [45]. While essential for initial repair, excessive fibrosis generates non-contractile scar tissue, provoking ventricular dilation and wall thinning that ultimately impairs cardiac function. Effective anti-fibrotic intervention is therefore critical for functional recovery [58]. To evaluate the anti-fibrotic effects of the hydrogel treatment, tissue samples from the infarcted region were collected 28 d post-treatment for histological analysis.

**Fig. 8:**
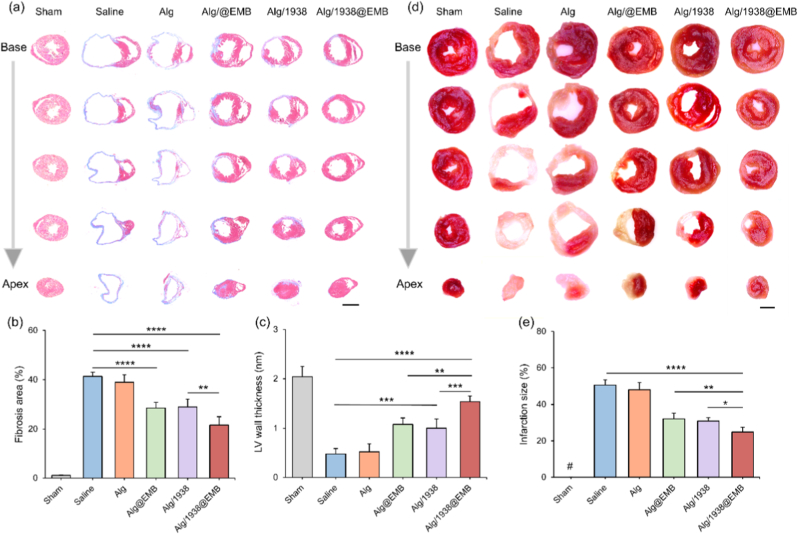
The effects of Alg/1938@EMB hydrogel on infarction size and fibrosis at Day 28 post-MI. (a) Representative Masson's trichrome-stained sections at corresponding levels (scale bar: 2 mm) and Quantification of (b) fibrosis area and (c) LV wall thickness (c). (d) TTC-stained sequential heart sections (scale bar: 2 mm). (e) Infarct size expressed as a percentage of the LV area in hydrogel-treated versus MI control mice, based on TTC staining. # indicates no detectable signal. Data are mean ± SD (*n* = 5; ∗*p* < 0.05, ∗∗*p* < 0.01, ∗∗∗*p* < 0.001, ∗∗∗∗*p* < 0.0001).

Masson's trichrome staining assessed fibrosis and ventricular wall thickness (Fig. 8a). Saline (MI control) and Alg groups exhibited extensive infarction with wall collapse, thinning, and prominent blue collagen deposition (fibrosis areas: 41.3 ± 1.7 % vs. 39.0 ± 2.9 %). Conversely, Alg@EMB and Alg/1938@EMB groups showed reduced fibrosis and more viable tissue. Quantitative analysis (Fig. 8b) revealed significantly smaller infarcts in Alg@EMB (28.5 ± 2.4 %) and Alg/1938 (28.9 ± 3.1 %) groups, with Alg/1938@EMB achieving maximal reduction (21.6 ± 3.4 %). As shown in Fig. 8c, the left ventricular (LV) wall thickness increased from 0.48 ± 0.11 mm (Saline) to 1.54 ± 0.11 mm (Alg/1938@EMB), confirming the composite hydrogel's anti-fibrotic and structural preservation effects. In addition, infarct size quantification via TTC staining was performed, and infarct areas were quantified as a percentage of the total LV area. As shown in Fig. 8d–f, Alg treatment (48.1 ± 4.0 %) provided no significant protection versus Saline (50.6 ± 2.8 %), while BMP-9-loaded hydrogels significantly reduced infarct size (Alg@EMB: 32.1 ± 3.2 %; Alg/1938@EMB: 24.8 ± 2.6 %). The Alg/1938 group also showed a significant reduction in infarct size (30.9 ± 1.9 %) versus Saline group, underscoring the cardiacprotection effect of UCL-TRO-1938. Notably, the Alg/1938@EMB group exhibited the greatest reduction in infarct size, reflecting synergistic actions of UCL-TRO-1938-enhanced vascularization coupled with BMP-9-mediated fibrotic suppression.

#### 3.5.4 Restoring heart function

**Fig. 9:**
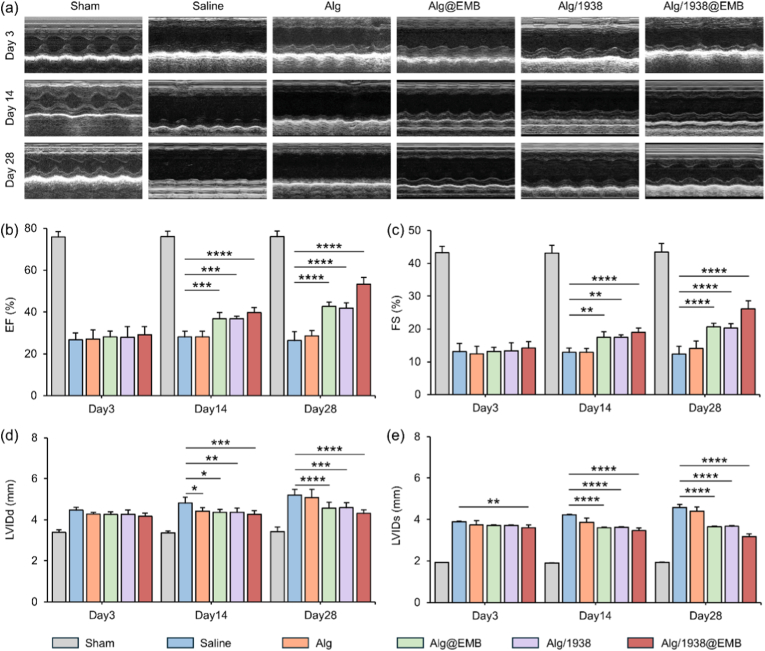
Effect of Alg/1938@EMB hydrogel on the cardiac function evaluated by echocardiography. (a) Representative images of M-mode echocardiography at Day 3, Day 14, and Day 28 post MI; (b, c) Quantitative analysis of (b) EF, (c) FS, (d) LVIDd, and (e) LVIDs at the indicated time points. Data are mean ± SD (*n* = 5; ∗*p* < 0.05, ∗∗*p* < 0.01, ∗∗∗*p* < 0.001, ∗∗∗∗*p* < 0.0001).

Echocardiography assessed cardiac functional parameters including ejection fraction (EF), fractional shortening (FS), and left ventricular internal diameters at end-diastole (LVIDd) and end-systole (LVIDs) (Fig. 9a). EF and FS indicate left ventricular contractility and global systolic function, while LVIDd and LVIDs reflect ventricular volume and geometric changes during cardiac cycles.

All parameters in Sham group remained within normal physiological ranges. Post-MI, the Saline group showed marked EF decline from 75.9 ± 2.6 % to 26.7 ± 3.3 %, indicating severe systolic dysfunction. The Alg group provided no significant improvement (EF: 28.6 ± 2.5 %), confirming alginate's limited efficacy [38]. Functionalized hydrogels preserved systolic function: EF increased to 42.7 ± 2.0 % (Alg@EMB), 41.9 ± 2.5 % (Alg/1938), and 53.4 ± 3.2 % (Alg/1938@EMB) (Fig. 9b), demonstrating synergistic cardiac recovery with UCL-TRO-1938 and BMP-9. FS measurements followed similar trends (Fig. 9c). Critically, Alg/1938@EMB significantly reduced LVID.d and LVID.s versus other MI groups, indicating attenuated ventricular dilation and preserved cardiac geometry (Fig. 9d and e).

Collectively, the composite hydrogel Alg/1938@EMB reduces apoptosis and inflammation, promotes angiogenesis, inhibits fibrosis and collagen deposition, increases left ventricular wall thickness, decreases infarct size, prevents cardiac dilation, improves systolic function, limits adverse remodeling, and enhances overall cardiac performance post-MI. These multifunctional effects demonstrate significant therapeutic advantages over single-component approaches (Alg@EMB or Alg/1938), establishing programmed dual-factor release as a robust strategy for post-MI cardiac recovery.

#### 3.5.5 Biosafety assessment

**Fig. 10:**
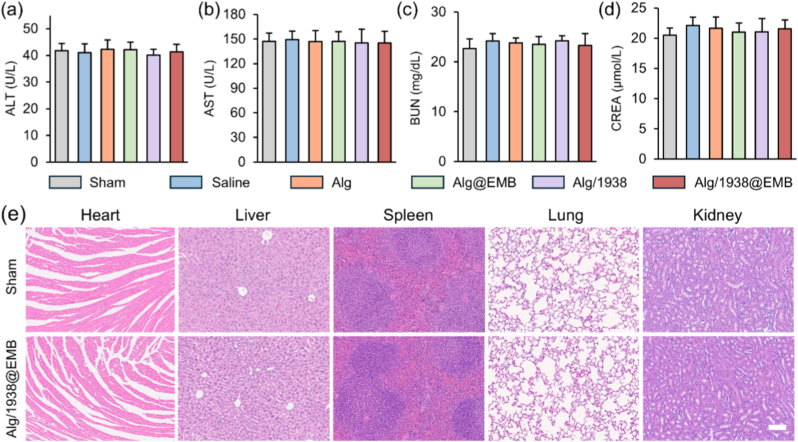
Biocompatibility evaluation of Alg/1938@EMB hydrogel. (a–d) Serum biochemical analysis of liver (ALT, AST) and kidney function markers (BUN, CREA); (e) H&E staining of major organs (scale bar: 100 μm). Data are mean ± SD (*n* = 5).

MSNs are extensively utilized in biomedical applications due to their favorable biocompatibility, structural stability, and established safety profiles [59]. Alginate-based injectable hydrogels have advanced to Phase I/II clinical trials, underscoring their translational potential and clinical safety [60]. To comprehensively evaluate the biocompatibility and immunogenicity of nanoparticle-loaded composite hydrogels, we assessed systemic toxicity and histopathological changes 28 d post-intramyocardial administration. Serum concentrations of hepatic injury markers (alanine aminotransferase [ALT], aspartate aminotransferase [AST]) and renal function indicators (blood urea nitrogen [BUN], serum creatinine [CREA]) were quantified. No significant differences occurred between treatment groups and sham controls across all parameters (Fig. 10a–d), confirming absence of hepatic or renal toxicity. Histological examination of major organs (heart, liver, kidneys, spleen, lungs) via H&E staining revealed no abnormal morphology, inflammatory infiltration, or damage (Fig. 10e). These results confirm the Alg/1938@EMB hydrogel's excellent biosafety and low immunogenicity, supporting its translational feasibility.

## 4 Conclusion

This study establishes UCL-TRO-1938 as a novel pro-angiogenic small-molecule agent for myocardial infarction therapy and develops an injectable composite Alg/1938@EMB hydrogel enabling spatiotemporally coordinated delivery of UCL-TRO-1938 and BMP-9. Through sequential promoting angiogenesis via rapid UCL-TRO-1938 release and inhibiting fibrosis via delayed BMP-9 release, this dual-delivery system mimicked natural post-MI healing progression. The composite hydrogel demonstrates enhanced efficacy beyond single-factor interventions, improving neovascularization, reducing apoptosis and fibrosis, and restoring cardiac function in vivo. Critically, the injectable alginate platform combines minimally invasive application with stage-specific drug release aligned with pathological progression through stimuli-responsive design. These findings underscore the therapeutic potential of temporally programmed biomaterial systems for myocardial repair. Subsequent investigations will pursue large-animal validation and manufacturing scalability to advance clinical translation.

## CRediT authorship contribution statement

**Yujuan Jia:** Investigation, Writing – original draft. **Tongtong Yin:** Methodology. **Zhu Wang:** Methodology. **Lei Chen:** Validation. **Hongyou Fan:** Validation. **Jiangbin Wu:** Validation. **Qian Yu:** Writing – review & editing. **Yanxia Zhang:** Conceptualization, Project administration, Writing – review & editing. **Zhenya Shen:** Funding acquisition, Supervision.

## Declaration of competing interest

The authors declare that they have no known competing financial interests or personal relationships that could have appeared to influence the work reported in this paper.

## Data Availability

Data will be made available on request.
