# Supplementary material for: Injectable alginate composite hydrogel with spatiotemporal codelivery of pro-angiogenic and anti-fibrotic agents for synergistic myocardial repair
Source: Mater Today Bio. 2026 Jan 28;37:102854. doi: 10.1016/j.mtbio.2026.102854 (PMC12887425; doi:10.1016/j.mtbio.2026.102854)
Supplement: Multimedia component 1 [file mmc1.pdf]

Supporting Information

Injectable Alginate Composite Hydrogel with Spatiotemporal Codelivery of Pro-Angiogenic and Anti-Fibrotic Agents for Synergistic Myocardial Repair

Yujuan Jia[a,c], Tongtong Yin[a], Zhu Wang[a], Lei Chen[a], Hongyou Fan[a], Jiangbin Wu[a], Qian Yu[b,*], Yanxia Zhang[a,*], Zhenya Shen[a,*]

[a] Department of Cardiovascular Surgery of the First Affiliated Hospital & Institute for Cardiovascular Science, Suzhou Medical College of Soochow University, Soochow University, Suzhou, 215006, China

[b] State and Local Joint Engineering Laboratory for Novel Functional Polymeric Materials, College of Chemistry, Chemical Engineering and Materials Science, Soochow University, Suzhou, 215123, China

[c] Department of Cardiovascular Surgery of the Qinghai Provincial People's Hospital, Xining 810000, China

*E-mail addresses: yuqian@suda.edu.cn (Q. Yu), zhangyanxia@suda.edu.cn (Y. Zhang), uuzyshen@aliyun.com (Z. Shen)

S1 Materials and Methods

S1.1 Materials

Sodium alginate, calcium gluconate, and zinc chloride ($ZnCl_2$) were obtained from Sigma-Aldrich (USA). Amine-modified mesoporous silica nanoparticles ($MSN-NH_2$) were purchased from Jike Biotechnology (China). The EdU cell proliferation assay Click-iT™ EdU Imaging Kit, 4',6-diamidino-2-phenylindole (DAPI), and TUNEL apoptosis detection kit were obtained from Beyotime Biotechnology (China). The Cell Counting Kit-8 (CCK-8) was purchased from Vazyme Biotech (China). TRIzol reagent was obtained from Invitrogen (USA). PrimeScript™ RT Master Mix and TB Green® Premix Ex Taq™ II were purchased from Takara Bio (Japan). Recombinant human transforming growth factor-β (TGF-β), and anti-α-SMA antibody were purchased from Proteintech (USA). Anti-CD31 antibody was obtained from Cell Signaling Technology (USA). Alexa Fluor 488-conjugated anti-IgG (H+L) secondary antibody and Alexa Fluor 594-conjugated anti-IgG (H+L) secondary antibody were obtained from Abcam (UK). UCL-TRO-1938 was purchased from Cayman Chemical (USA). Recombinant human BMP-9 was acquired from R&D Systems, Bio-Techne (USA), and the BMP9 ELISA kit was purchased from LiankeBio (China). The Masson's trichrome staining kit and Hematoxylin-Eosin (H&E) Staining Kit were sourced from Solarbio (China). 2% TTC Staining Solution was obtained from Biosharp (China). Male C57BL/6 mice were procured from Suzhou Zhaoyan (China).

S1.2 Characterization of EBM NPs

Particle morphology was observed via SEM (Hitachi S4800) and TEM (Hitachi HT7700). Size and zeta potential were measured using a Zetasizer Nano ZS90. Elemental distribution was examined using EDS coupled with FESEM (Hitachi S4700).

For scanning electron microscopy (SEM, Hitachi S4800), nanoparticle suspensions (1 mg/mL in DI water) were sonicated for 2 min (100 W, ice bath) and drop-cast onto silicon wafers. After vacuum-drying overnight, samples were sputter-coated with 5-nm gold/palladium (Quorum Q150T ES) and imaged at 15 kV accelerating voltage in secondary electron mode. Transmission electron microscopy (TEM, Hitachi HT7700) analysis utilized samples prepared by depositing ethanol-diluted suspensions (0.01 mg/mL) onto 300-mesh carbon-coated copper grids, followed by 30-s probe sonication (40% amplitude) and blotting of excess liquid. Grids were air-dried for 24 h prior to imaging at 70 kV. Hydrodynamic size distribution and zeta potential were measured using a Zetasizer Nano ZS90 (Malvern Panalytical). Nanoparticles dispersed in 10 mM PBS (pH 7.4) at 0.1 mg/mL underwent 3-min bath sonication (Branson 2510), with triplicate measurements at 25°C (detection angle: 173°). Elemental mapping employed energy-dispersive X-ray spectroscopy (EDS) coupled with field-emission SEM (FESEM, Hitachi S4700). Samples prepared identically to SEM analysis were scanned at 15 kV (60-s live time/mapping region), with elemental profiles processed via INCA Suite v4.15.

S1.3 Characterization of the Alg/1938@EMB hydrogel

S1.3.1 SEM

Hydrogel samples were frozen in liquid nitrogen for 60 s and lyophilized at −60°C under a 0.05 mbar vacuum for 48 h. The freeze-dried hydrogels were carefully fractured to expose their internal microstructure, mounted on aluminum stubs, and sputter-coated with a 10-nm Au/Pd layer. The morphology was then examined using field-emission scanning electron microscopy (FE-SEM; Hitachi SU8010) at an accelerating voltage of 5 kV in secondary electron mode.

S1.3.2 Injectability Assessment

The injectability of freshly prepared hydrogels was evaluated by extruding them through a 30-gauge microneedle (inner diameter: 0.16 mm) at a constant displacement rate of 5 mm/min using a mechanical testing system.

S1.3.3 Rheological Characterization

For steady-state rheological analysis, 500 μL of hydrogel was loaded between 25-mm parallel plates with a 0.5 mm gap on a rotational rheometer (AR2000, TA Instruments, USA). Samples were equilibrated at 37°C for 5 min prior to testing. Shear rate sweeps were performed from 0.3 to 2.0 s^{-1} to assess viscosity profiles. Dynamic mechanical properties were evaluated by monitoring the storage modulus (G') and loss modulus (G'') for 300 s at a frequency of 1 Hz and 1% strain (within the linear viscoelastic region), maintaining a temperature of 37.0 °C.

S1.4 *In Vitro* cell viability assessment

HUVECs, NIH3T3 fibroblasts, and HL-1 cardiomyocytes were seeded in 96-well plates and cultured at 37 °C with 5% CO_2 for 24 h. The medium was then replaced with fresh medium containing 10 μL of the following treatments: PBS, Alg, Alg@MSN, Alg@MB, Alg@EMB, Alg/1938@EMB. Cell viability was assessed at 24, 48, and 72 h post-treatment using the CCK-8 assay. After incubation with 10% CCK-8 reagent for 2 h, absorbance at 450 nm was measured.

S1.5 Quantitative Real-Time PCR (qRT-PCR)

Total RNA was extracted from HUVECs after hypoxic treatment using TRIzol, and cDNA was synthesized using PrimeScript™ RT Master Mix (Takara). qRT-PCR was performed using TB Green® Premix Ex Taq™ II on a BIOER system. Gene expression was normalized to 18S rRNA and calculated using the $2^{-}\Delta\Delta Ct$ method. Primer sequences are listed in **Table S1**. All reactions were run in triplicate.

Table S1. Sequences of the primers in qRT-PCR

Target Gene (Human)	Primer	Sequence (5′–3′)
VEGFA	Forward	AGGGCAGAATCATCACGAAGT
	Reverse	AGGGTCTCGATTGGATGGCA
PECAM1	Forward	AACAGTGTTGACATGAAGAGCC
	Reverse	TGTAAAACAGCACGTCATCCTT
NOS3	Forward	TGATGGCGAAGCGAGTGAAG
	Reverse	ACTCATCCATACACAGGACCC
PIK3CA	Forward	CCACGACCATCATCAGGTGAA
	Reverse	CCTCACGGAGGCATTCTAAAGT
AKT1	Forward	AGCGACGTGGCTATTGTGAAG
	Reverse	GCCATCATTCTTGAGGAGGAAGT
S6KB1	Forward	CGGGACGGCTTTTACCCAG
	Reverse	TTTCTCACAATGTTCCATGCCA
18S	Forward	GTAACCCGTTGAACCCCATT
	Reverse	CCATCCAATCGGTAGTAGCG

S2 Supporting Results

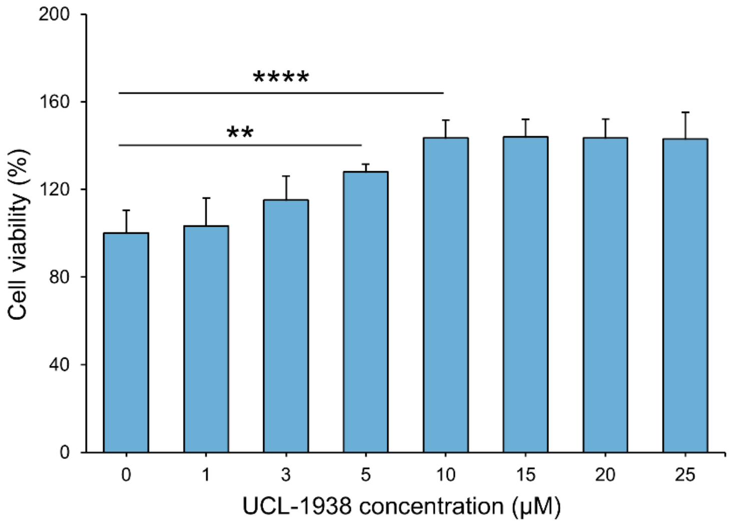

Figure S1. The effects of different concentrations of UCL-TRO-1938 on HUVECs under hypoxic conditions. Data are presented as mean ± SD (n = 5. $^{**}p < 0.01$, $^{****}p < 0.0001$).

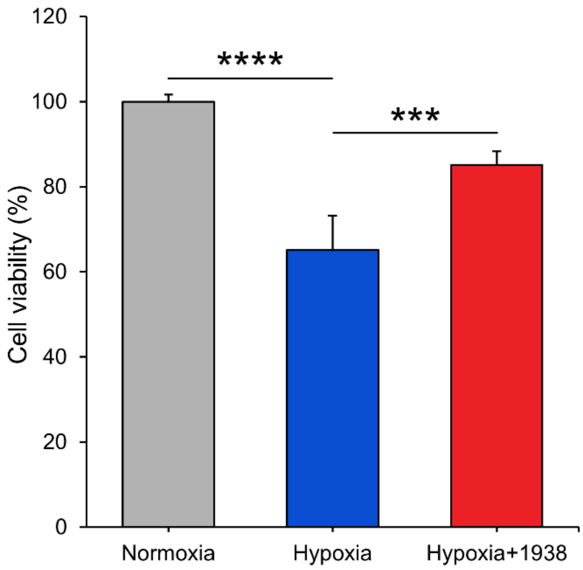

Figure S2. Protective effects of UCL-TRO-1938 on HUVECs under hypoxic conditions. Data are presented as mean ± SD (n = 5. $^{***}p < 0.001$, $^{****}p < 0.0001$).

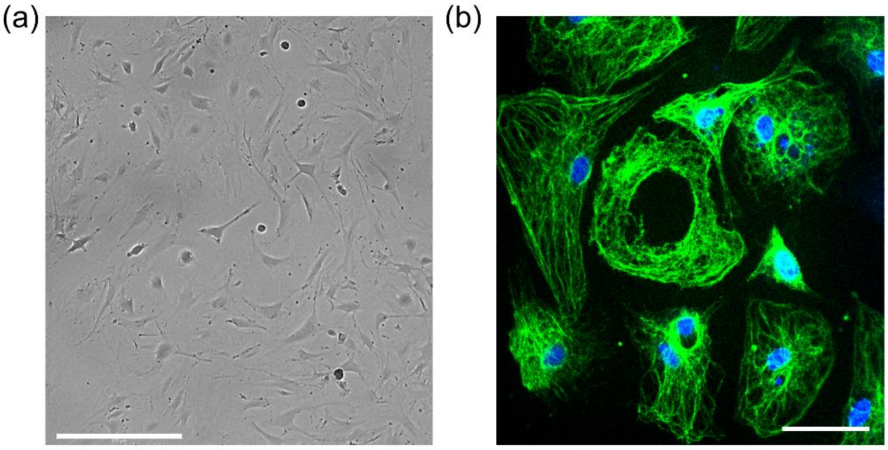

Figure S8. Morphological observation and immunofluorescence characterization of neonatal mouse cardiac fibroblasts (CFs). (a) Phase-contrast micrograph of primary CFs. (Scale bar: 275 μm.) (b) Immunofluorescence staining confirming the identity and purity of the isolated CFs. The purity of CFs (%) was calculated as the number of nuclei within green-labeled cytoplasm divided by the total number of nuclei, multiplied by 100%, based on fluorescence imaging. Nuclei were stained with DAPI (blue), and Vimentin was labeled in green. (Scale bar: 50 μm.)

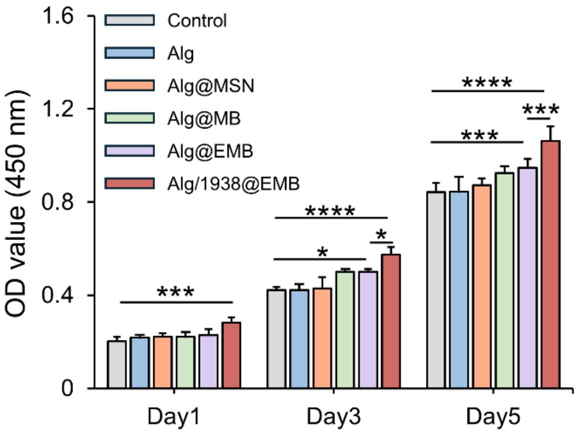

Figure S4. Effects of different hydrogels on HUVEC viability assessed by CCK-8 assay. Data are presented as mean ± SD (n = 5. $^{*}p < 0.05$, $^{***}p < 0.001$, $^{****}p < 0.0001$).

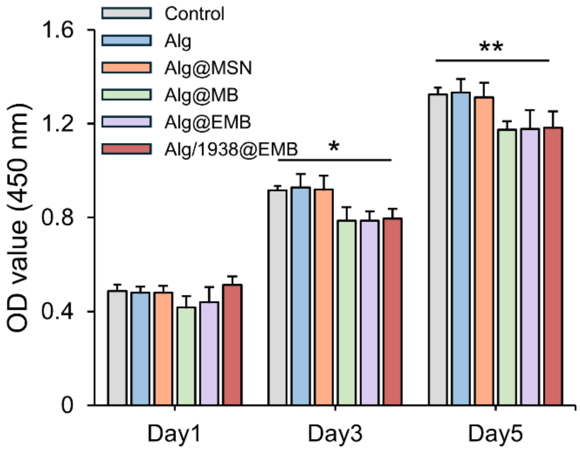

Figure S5. Effects of different hydrogels on NIH3T3 viability assessed by CCK-8 assay. Data are presented as mean ± SD (n = 5. $^{*}p < 0.05$, $^{**}p < 0.01$).

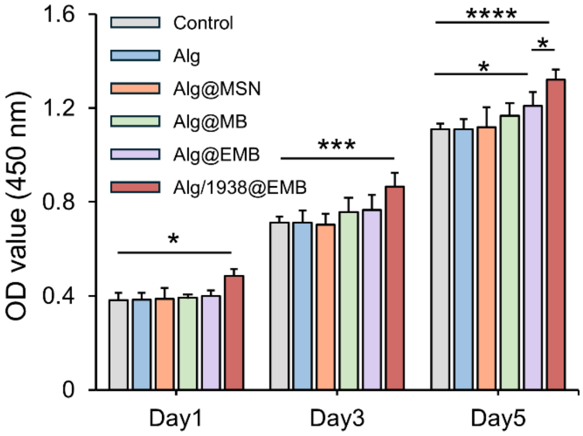

Figure S6. Effects of different hydrogels on HL-1 cells viability assessed by CCK-8 assay. Data are presented as mean ± SD (n = 5. $^{*}p < 0.05$, $^{***}p < 0.001$, $^{****}p < 0.0001$).

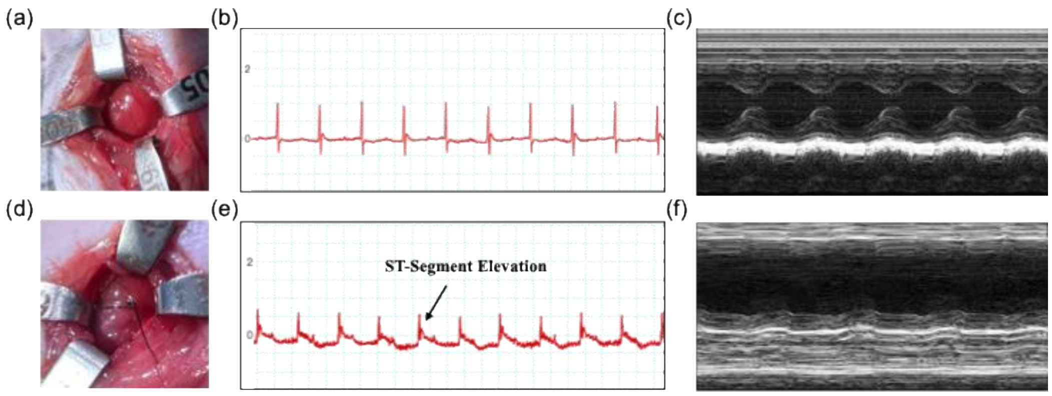

Figure S7. Establishment and validation of the murine MI model. (a) Representative image of a heart from the Sham group without coronary artery ligation. (b) Representative electrocardiogram (ECG) tracing from a sham-operated mouse. (c) Echocardiographic image of a heart from the Sham group. (d) Representative image of an infarcted heart following coronary artery ligation. (e) Representative ECG tracing post-MI, showing characteristic ST-segment elevation (indicated by the black arrow). (f) Echocardiographic image of a heart post-MI, demonstrating impaired contractility.

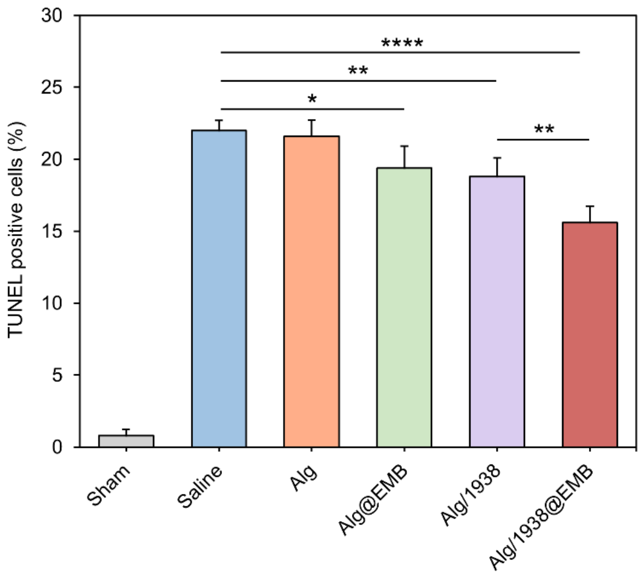

Figure S8. Quantitative results of TUNEL-positive cell ratio. Data are presented as mean ± SD ($n = 5$. $^{*}p < 0.05$, $^{**}p < 0.01$, $^{****}p < 0.0001$).
